# A mixed-methods study of emotional support for families of organ donors in Hunan Province, China

**DOI:** 10.3389/fpsyg.2022.952524

**Published:** 2022-09-02

**Authors:** Wenzhao Xie, Shufeng Kong, Haiyan He, Huan Xiong, Qizhen Zhu, Panhao Huang

**Affiliations:** ^1^The Third Xiangya Hospital of Central South University, Changsha, China; ^2^Key Laboratory of Medical Information Research (Central South University), College of Hunan Province, Changsha, China; ^3^School of Life Sciences, Central South University, Changsha, China; ^4^School of Medical Information Engineering, Jining Medical University, Rizhao, China; ^5^Department of Pharmacy, The Third Xiangya Hospital, Central South University, Changsha, China

**Keywords:** organ donation, donor families, mental health, emotional support, coping styles

## Abstract

**Background:**

Family consent is a prerequisite for the organ donation of the deceased in China. However, a large number of donors are individuals who died due to accidental injuries or unanticipated diseases, which means that most of the families of such donors have just experienced the sudden death of their loved one and have to make a donation decision in a short time. This decision may cause psychological stress and some psychological damage to the minds of relatives of the donors. In addition, cultural sensitivity also has largely caused the relatives of donors inner conflicts and contradictions. And sometimes organ donation may still be stigmatized. However, have they received any emotional support and what is their emotional support needs are some questions that need to be answered. Therefore, this study aims to investigate the emotional support, influencing factors, and needs of the family members of organ donors in Hunan Province, China.

**Materials and methods:**

This is mixed-methods research that combines quantitative and qualitative research methods. A cross-sectional survey was conducted among 102 donor families using a questionnaire to investigate their emotional support status. To further understand their emotional support needs, 12 donor families participated in the semi-structured interview.

**Results:**

The results confirmed that: (1) A total of 67.7% of the 102 respondents received emotional support or psychological comfort. Thus, only a small number of respondents (31.4%) felt respected by the public. (2) Emotional support came mainly from immediate family members (73.91%), and official organizations such as the Red Cross (43.48%). (3) Marital status, health status, occupation, and coping style can affect the emotional support of the donor families (*p* < 0.05). (4) Interview showed that the families of donors need emotional support and psychological aid from psychological professionals mostly. And they also wish to receive the understanding and respect of the public.

**Conclusion:**

Most families of organ donors received emotional support from family, Red Cross, and friends, but only a minority of families of donors reported receiving respect from the public after the donation. And families of donor showed a strong need for emotional support and professional psychological aid from institutions.

## Introduction

Organ transplantation has been called the pinnacle of twenty-first century medicine, bringing hope for a cure to many patients with end-stage organ failure. However, growing shortages of organ are an obstacle to organ transplants in many countries (Ma et al., 2021). For this reason, the WHO, the International Society for Transplantation (TTS), and the International Society for Organ Donation and Access (ISODP) have jointly developed a strategic plan for organ donation after a person’s death. According to statistics, approximately 120,000 organ transplants are performed and completed worldwide each year, with more than 75% of the donors being deceased individuals. Since 1 January 2015, China has completely stopped using the organs of deceased prisoners as a source of transplant donors and has promoted voluntary organ donation. As of 26 February 2022, according to the China Organ Donation Administrative Center (2022), there were a total of 38,681 organ donation cases, and 116,092 large organs of various types had been donated, saving the lives of more than 110,000 patients. The top four countries in the world that perform organ donation and transplantation are the United States, China, Brazil, and Spain, accounting for more than 60% of the total organ donation in the world (Jiang et al., 2021). Family consent is a prerequisite for organ donation of the deceased in most countries around the world (Knhis et al., 2021). Organ donation is a “distressing experience.” Because the “donor” is deceased at the time of donation, this psychological pain is transferred to the family of the donor (Ahmadian et al., 2020). In clinical practice, a large number of donors are individuals who died due to accidental injuries or unanticipated diseases, which means that most of the families of donors have just experienced the sudden death of their loved one and have to make a donation decision in a short time. The short-time decisions may cause psychological stress and some psychological damage, which could lead to distress, depression, anxiety, posttraumatic stress disorder, and other psychological disorders leading to serious cases (Tirgari et al., 2020; Peng et al., 2021). Lopez et al. (2018) proposed an integrated psychological model for the relatives of the organ donor for a better decision making (IMROD). Wind et al. (2022) pointed out that the care of organ donor families is an important part of the organ donation process.

At present, organ donors in China are roughly distributed in the age range of 20–50 years, and they are an important economic pillar of their families, and their families may face multiple economic and psychological crises after their sudden illness or unexpected death and organ donation (Shi et al., 2020). The traditional belief of “preserving the integrity of the body” (Li et al., 2019; Nie and Jones, 2019) has also led to a psychological burden for donors’ families. Our group’s previous study also showed that the concept of “completion of the body” is an important factor, which influences the decision-making of donor families (Xie and Li, 2016). Cultural sensitivity has largely caused their inner conflicts and contradictions. In addition, though the national and local governments have vigorously publicized and encouraged organ donation in China, celebrating this selflessness and love, organ donation may still be stigmatized (Zhang and Feng, 2019). News reports have mentioned that some families of organ donors in financial difficulties who have received certain material assistance from the Red Cross are considered to have sold the child’s organs for money (Chen, 2021). Therefore, organ donation may also make the donor’s family feel stressed or cause psychological problems. One survey showed that the prevalence of depression among families of organ donors in China is approximately 31%, and the prevalence of anxiety is 43% (Yang et al., 2018).

Humanitarian aid is the last step in the human organ donation workflow. Emotional support is an important part of organ donor family support, and it has a profound impact on preventing, controlling, and alleviating the psychological damage of the donor’s relatives, promoting their psychological recovery, and safeguarding their psychological health. Tirgari et al. (2020) found moderate levels of bereavement, depression, and PTSD among organ donor families in Iran. Some scholars have suggested that psychological crisis intervention should be conducted according to the different periods and characteristics of the donor’s family (Pan, 2019; Zhang and Feng, 2019).

Mixed-methods research is an approach that combines quantitative and qualitative research methods to collect and analyze data, integrate research findings. It can help to have an in-depth understanding of various interesting phenomena that cannot be fully understood using only a quantitative or a qualitative method (Schoonenboom and Johnson, 2017; Hong et al., 2019). Therefore, we used mixed methods to analyze the emotional support, influencing factors, and needs of the family members of organ donors in Hunan Province, China, which can be conducive to psychological intervention for the family members of organ donors in the next step. Yet it’s worth noting that organ donation in this article refers to voluntary organ donation after the death of citizens, excluding living organ donation by relatives and body donation.

## Materials and methods

### Research participants

The participants were immediate family members of organ donors in Hunan Province, such as spouses, parents, and children. The inclusion criteria were as follows: above the age of 18; direct family members of organ donors; good communication and reading ability; and voluntary participation.

### Research tools

The questionnaire consisted of three parts: general demographic information, an emotional support questionnaire for organ donors’ families, and the Simplified Coping Style Questionnaire.

(1) The general demographic information questionnaire included nine items: gender, age, marital status, education level, health status, occupation, income, household location, and kinship with the organ donor.
(2) The Simplified Coping Style Questionnaire was used to analyze the psychological stress, anxiety, social avoidance, and distress of the donor’s family. The Simplified Coping Style Questionnaire was revised based on the Ways of Coping Questionnaire compiled by Folkman. And the questionnaire was verified in the Chinese population by Xie (1998). It consisted of 20 items, of which 1–12 items formed the positive coping dimension, and 13–20 items formed the negative coping dimension. If the coping style score is greater than 0, the subjects mainly adopt a positive coping style under stress, but if it is less than 0, the subjects are accustomed to negative coping style The research results showed a significant relationship between an individual’s coping style and mental health (Xie, 1998; Dai, 2010).
(3) The Emotional Support Questionnaire for Families of Organ Donors was compiled by the research group, then discussed by the research group many times, reviewed by experts, and revised based on a pilot survey. It was divided into two parts, namely, support and demand, and had seven items, mainly in the form of multiple-choice questions: ➀ As a family member of a donor, have you ever received emotional support or spiritual comfort from others or organizations? ➁ What is the main source of emotional support you have received? ➂ Have you been respected by the public as a donor’s family? ➃ Do you regret the decision you made to donate? ➄ If so, what caused you to regret your decision to donate? ➅ Would you like to receive moral comfort and emotional support from others or organizations? ➆ Do you want the state to establish a psychological counseling institution for organ donor families?

The qualitative component of this study used semi-structured interviews. The research team initially drew up an interview outline, which was revised after pre-interviews with two organ donor families to form a formal interview outline. The outline included three main topics: ➀ Did the event of organ donation causes any changes in your emotions over time? ➁ Have there been any people or organizations who have given you emotional support after your relative donated organs? What were the main ways? ➂ Do you feel that the emotional support you received was adequate? What other emotional support is needed?

### Data collection and analysis

#### Questionnaire distribution

Participation in the study was voluntary; the research team distributed 79 paper questionnaires to donor families and 33 electronic questionnaires through the Questionnaire Star platform. A total of 102 valid questionnaires were collected, and the effective rate was 91%. SPSS 23.0 was used for statistical analysis. The analysis methods used included the calculation of frequencies and percentages, the chi-square test, the rank-sum test, and multiple logistic regression, and the significance level was α = 0.05.

#### Semi-structured interviews

The sample size in qualitative research depends on whether the information obtained reaches saturation. If new interviewees continue to be included and no new information emerges, the information has reached saturation, and sampling stops. A total of 12 interviewees were eventually included in this study. During the interview, after obtaining the consent of the interviewees, we took sound recordings, and the typical behaviors and reactions of the interviewees were observed and recorded. The interview materials were jointly coded by two researchers. To protect the privacy of the respondents, the interviewees are numbered and presented in the form of “D1, D2, D3, D4, D5, D6, D7, D8, D9, D10, D11, and D12.” The results of the semi-structured interviews were mainly combined with the grounded theory analysis method. The qualitative analysis software NVivo 12.0 was used to assist in completing the thematic analysis. The data analysis was performed by three members of the research team who were familiar with qualitative coding.

### Ethics

Approval for the study was obtained from the Institutional Ethics Committee of The Third Xiangya Hospital, Central South University.

## Results

### Demographic data of respondents

The general demographic data of the respondents are shown in Table 1. There were 48 (47.06%) male family members and 54 (52.94%) female family members who participated in the study, with an average age of 42.2 ± 12.7 years. Of the 102 participants, 75 (73.5%) were married, and 56 (54.9%) were junior high school students or below. Forty-eight (47.1%) patients had general health conditions. The occupation was mainly farmers (40.2%). The average monthly income of 46 participants was less than 1,500 yuan, and 79 (77.5%) were rural residents. The survey was mainly conducted by the parents of the donors (47.46.1%).

**TABLE 1:** General demographic data of respondents (*N* = 102).

Variables	Frequency	Percentage (%)	Variables	Frequency	Percentage (%)
**Gender**			**Occupation**		
Male	48	47.1	Occupation^a^	20	19.6
Female	54	52.9	Occupation^b^	41	40.2
**Age (year)**			Freelance	17	16.7
≤25	9	8.8	Other	24	23.5
25–35	23	22.6	**Monthly income (CNY)**		
≥35	70	68.6	≤1,500	46	45.1
**Marital status**			1,500–5,000	41	40.2
Unmarried	12	11.8	≥5,000	15	14.7
Married	75	73.5	**Registered residence**		
Divorced	4	3.9	City	23	22.6
Widowed	10	9.8	Countryside	79	77.5
Other	1	1	**Relationship with donor**		
**Educational level**			Spouse	7	6.9
≤Junior high school	56	54.9	Parents	47	46.1
High middle school	39	38.2	Son or daughter	15	14.7
≥Undergraduate	7	6.9	Brother or sisters	22	21.6
**Health condition**			Grandfather	1	1
Poor	21	20.6	Grandmother	1	1
Average	48	47.1	Grandchildren	1	1
Good	33	32.4	Other	8	7.8

Occupation^a^: Government staff, business personnel, or technical staff.

Occupation^b^: Famers.

A total of 12 interviewees officially participated in this study. They were numbered D1, D2, and D12. The general demographic data of the interviewees are shown in Table 2. The interviewees were the fathers (8.67%), mothers (1.8%), sisters, and brothers (3.25%) of donors. Rural residents accounted for 58% of the respondents. Fifty-eight percent had junior high school education or less.

**TABLE 2:** General demographic data of the interviewees (*N* = 12).

Number	Relationship with the donor	Age (year)	Location	Education level
D1	Father	47	Countryside	Illiterate
D2	Elder sister	52	Countryside	Illiterate
D3	Father	67	City	Senior high school
D4	Father	54	Countryside	Junior high school
D5	Father	49	City	Undergraduate
D6	Father	47	Countryside	Junior high school
D7	Elder sister	50	City	Junior high school
D8	Father	52	City	Junior college
D9	Mother	34	Countryside	Junior high school
D10	Father	45	City	Junior college
D11	Father	41	Countryside	Junior high school
D12	Brother	35	Countryside	Junior college

### Results of the quantitative study

#### Results of the simple coping style questionnaire

The simple coping style questionnaire had good reliability and validity, with a Cronbach’s α coefficient of 0.80 and a KMO of 0.714. The mean scores of the positive coping subscale and the negative coping subscale for organ donor families were 1.65 ± 0.53 and 1.36 ± 0.55, respectively, with coping tendency values ranging from −2.06 to 2.80. Among these, 49 had a positive coping style, with a coping style value greater than 0, and 53 had a negative coping style.

#### Current status of emotional support for donor families

The emotional support questionnaire of organ donor families is shown in Table 3. A total of 67.7% of the 102 respondents received emotional support or psychological comfort. Only a small number of respondents (31.4%) felt respected by the public. A few respondents regretted agreeing to donate (9.8%).

**TABLE 3:** Emotional support questionnaire of the donor’s families (**N** = 102).

Variables (entries)	Frequency	Percentage (%)
**Have you received emotional support**		
Yes	69	67.7
No	33	32.4
**Have you been respected by the public as a donor’s family**		
Yes	32	31.4
No	25	24.5
Uncertain	45	44.1
**Do you regret the decision you made to donate**		
No	12	11.8
Yes	10	9.8
Uncertain	28	27.5
Respect the donor’s decision	52	51.0

**FIGURE 1:**
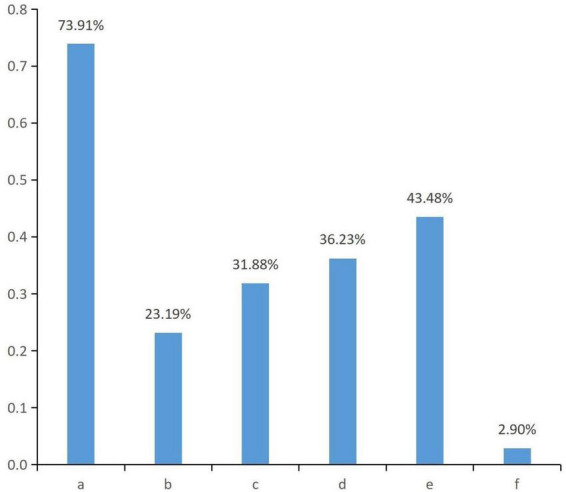
Access to sources of emotional support. a. Immediate family members; b. Other relatives; c. Friends, colleagues; d. Organ donor coordinators; e. Official organizations, such as government, Red Cross, etc.; f. Unofficial organizations, such as religious and social groups.

**FIGURE 2:**
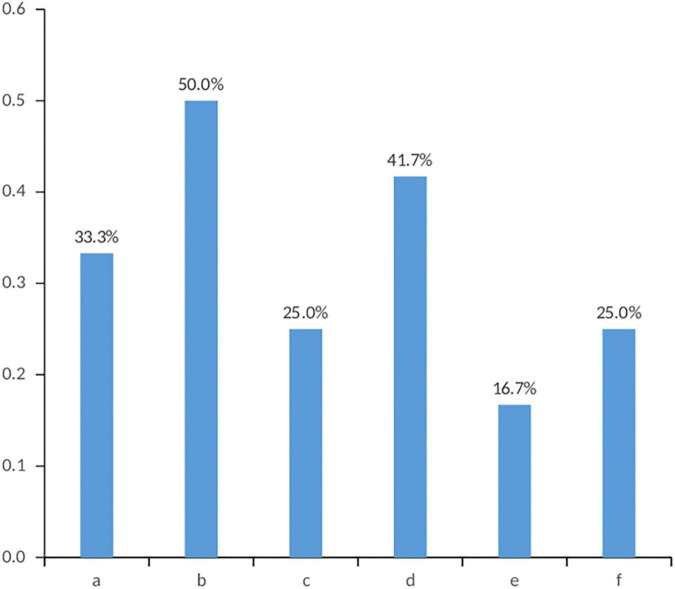
Reasons why family members regret agreeing to donate organs. a. People’s words; b. Lack of understanding by others; c. Opposition from other family members; d. Shame on the donor. e. The initial decision to donate was not well thought out; f. The community’s help after the donation did not meet expectations.

Emotional support came mainly from immediate family members (73.91%), official organizations such as the Red Cross (43.48%), and organ donation coordinators (36.23%) (Figure 1). Among the respondents who reported regretting donation, the reasons for regret were mainly not being understood by others (50.0%) and the shame of the donor (41.7%) (Figure 2).

#### Influencing factors of emotional support of donor families

(1) Taking whether the respondents received emotional support and the sources of emotional support as dependent variables and the eight demographic characteristics and coping style as independent variables, the chi-square test was used for univariate analysis (see Table 4). The results showed that marital status was statistically significantly (*p* = 0.020) related to donor families’ access to emotional support.

**TABLE 4:** Variance analysis of emotional support of respondents (*N* = 102).

Variables	Have you received emotional support		*χ^2^*	*p*
	No	Yes		
**Marital status**				
Unmarried	6 (50.0)	6 (50.0)	11.683	0.020*
Married	20 (26.7)	55 (73.3)		
Divorce	0 (0.0)	4 (100.0)		
Widowed	7 (70.0)	3 (30.0)		
Other	0 (0.0)	1 (100.0)		

**Variables**	**Support from government, Red Cross**		***χ^2^***	***p***
	**No**	**Yes**		

**Health condition**				
Poor	5 (29.4)	12 (70.6)	7.648	0.022*
Average	20 (71.4)	8 (28.6)		
Good	14 (58.3)	10 (41.7)		
**Coping style**				
Negative	27 (71.1)	11 (28.9)	7.267	0.007**
Positive	12 (38.7)	19 (61.3)		

**Variables**	**Support from friends and colleagues**		***χ^2^***	***p***
	**No**	**Yes**		

**Occupation**				
Farmer	23 (82.1)	5 (17.9)		
Other Professionals	24 (58.6)	17 (41.5)	4.269	0.039*

Statistically significant (*p* < 0.05)*. Statistically significant (*p* < 0.01)**.

Regarding emotional support provided by the government and Red Cross, 70.6% of those with poor health received support, 28.6% of those with fair health received support, and 41.7% of those with good health received support; the difference was statistically significant (*p* = 0.022). In addition, 61.3% of family members with positive coping style received support from the government and the Red Cross, while 28.9% of those with negative coping style received support, with a statistically significant difference (*p* = 0.007). Families with poorer health and positive coping received more emotional support from official organizations.

Regarding the emotional support provided by friends and colleagues, 17.9% of farmers received support, while 41.5% of other professionals received support. Family members with other occupations had higher rates of emotional support from friends and colleagues, and the difference was statistically significant (*p* = 0.039).

(2) For further analysis, the options of the entries were divided into 0–8 (up to eight items) according to the number of sources of obtained emotional support. Taking the degree of emotional support obtained by the subjects as the dependent variable and the eight demographic characteristics and coping style of the subjects as independent variables, a rank-sum test analysis was carried out. The results showed that family members with positive coping style received emotional support from multiple sources, and the difference was statistically significant (*p* = 0.034).

(3) To further clarify the effect and trend of various factors on family members’ emotional support, a multiple logistic regression analysis was conducted. The indicators with statistically significant differences in the univariate analysis were introduced into the logistic regression model one by one to identify the influencing factors of emotional support. The odds ratio (OR) and its 95% confidence interval (CI) were used to estimate each protective and risk factor. The test level was α in = 0.05 and α out = 0.1. The results are shown in Table 5.

**TABLE 5:** Logistic regression analysis of influencing factors on emotional support.

	*B*-value	SD	χ^2^	*p*	OR	95% CI
Constant^a^	−0.935	0.293	10.176	0.001**		
Other professionals (excluding farmers)	−0.591	0.293	4.056	0.044*	0.307	0.097−0.969
Constant^b^	0.051	0.283	0.032	0.857		
Positive coping style	0.884	0.304	8.473	0.004**	0.171	0.052−0.561

Constant^a^: the emotional support from friends, colleagues, etc.

Constant^b^: the emotional support from official organizations such as the Red Cross.

SD, Standard deviation.

Statistically significant (*p* < 0.05)*. Statistically significant (*p* < 0.01)**.

The results showed that having other occupations was a protective factor for emotional support from friends and colleagues compared to having an occupation as a farmer (OR: 0.307, 95% CI 0.097–0.969), and families with other occupations received more emotional support from friends and colleagues. Having positive coping style was a protective factor for emotional support from official organizations compared to having negative coping style (OR: 0.171, 95% CI 0.052–0.561), and families with positive coping style received more emotional support from official organizations.

#### Analysis of the emotional support needs of organ donor families

The emotional support needs of organ donor families are shown in Table 6. The results showed that 68.6% of the families needed spiritual comfort and emotional support. When asked whether they wanted the state to establish a psychological counseling institution for organ donor families, most of the families (77.5%) indicated that they did.

**TABLE 6:** Emotional support needs of the donor’s families (*N* = 102).

Variables	Frequency	Percentage (%)
**Would you like to receive moral comfort and emotional support from others or organizations?**		
Yes	70	68.6
No	17	16.7
Uncertain	15	14.7
**Do you want the state to establish a psychological counseling institution for organ donor families?**		
Yes	79	77.5
No	5	4.9
Uncertain	18	17.7

The results were analyzed by the chi-square test; whether families needed spiritual comfort and emotional support and whether families wanted the state to establish a psychological counseling institution for organ donor families were the dependent variables, and the eight demographic characteristics of the respondents and the scores of their coping style were the independent variables. The results showed that the demand rates for the establishment of psychological counseling institutions in families with average and good health were 85.4 and 78.8%, respectively. For those in poor health, the demand rate was 57.1%. The difference was statistically significant (*p* = 0.045), while the other variables were not statistically significant. This indicated that the demand for psychological aid agencies was higher among the healthier family members.

### Results of the qualitative study

The interview data were organized to distill and summarize the relevant themes from three aspects: access to emotional support, emotional support needs, and psycho-emotional aspects of organ donor families, as follows:

**Theme 1: Emotional support acquisition**

(1) Sources of emotional support

Among the families interviewed, nine families received care from family members, as well as friends, neighbors, and colleagues. Seven families received care and support from official organizations, such as the Red Cross, local governments and organ donation coordinators.

D1: *After the donation, relatives, friends, and classmates of the son visited the home. They mainly came to visit and show their care by persuasion, companionship, and dispersal. Many people came to the home to offer comfort*.

D2: *At that time, it was mainly the family who did the ideological work; everyone in the family approved of the donation, and the parents supported it. The organ donor Coordinator, Red Cross staff and local officials visited once, and relatives, friends, neighbors visited the home many times.*

(2) Forms of emotional support

The main forms of emotional support and psychological comfort for the families of organ donors were home visits, telephone calls to offer condolences, vacations, organized activities and companionship during the donation period from family members, friends, neighbors, organ donation coordinators, the Red Cross, and the local government, etc.

D1: *My siblings and my son’s classmates persuaded me, and my brother-in-law took me out for a break*.

D2: *The organ donation coordinator informed me about the group activities, and there was a phone call to greet me at the tomb visit every year*.

D3: *Friends and colleagues are concerned about me. After the donation, those who knew came to visit and offer comfort. I am grateful to the Red Cross, which was with me in the hospital for more than 20 days. During that time in the hospital, a lot of psychological support was given, and we could visit our son every day through the green channel. The organ donation coordinator often called us to ask if we were okay, and the local officials were more concerned, and they made phone calls on New Year’s Day to express their greetings.*

D5: *Most of the family members are supportive because they are more educated.*

D9: *The family gave psychological comfort after the donation, and the Red Cross staff also called to ask about the recent situation and express their concern.*

**Theme 2: Emotional support needs**

(1) Sources of emotional support needs

Seven family members showed that they need the emotional support of family members and friends most. Four families also wanted care and visits from official organizations.

D1: *I hope for and welcome relatives, friends, coordinators, and officials to visit me, and I will be very happy and warmly entertained if they come. I hope the state will establish a psychological counseling institution and more effectively communicate with professional people.*

D2: *Mothers need the most spiritual support, and it is better for professionals to give psychological guidance.*

(2) Forms of emotional support needs

The main emotional support needs were home visits, companionship, telephone condolences, participation in groups, and the establishment of psychological de-escalation institutions. Families invariably expressed the hope that the state would establish a psychological counseling institution for donor families to relieve psychological stress through communication with professionals. Four families explicitly said that they did not need staff to visit them at home. They did not want the sad event to be brought up, and telephone communication was fine.

D2: *The matter has passed. There is no need to call often to express condolences, and there is no need for staff to visit the family. We do not want to be disturbed and do not want to evoke sadness.*

D4: *At present, the spiritual care given is not enough. In the 6 months and 1 year after the donation, the family is very unstable emotionally and mentally. I hope that the Red Cross staff, social volunteers, and so on can make home visits, visits or calls during festivals to give psychological comfort. I hope that the state will establish a psychological counseling institution, which is very important.*

*D7: I really wanted to get more emotional support from relatives and friends. I hope the state will set up a psychological assistance organization.*

*D9: I need the support of my family most. My daughter was buried far away, and I feel bad when I come to visit the grave and remember what happened before. It would be good to establish a special psychological aid agency to help with psychological support.*

*D10: The state can establish relevant psychological aid institutions to give psychological support. More emotional support is desired.*

(3) Content of emotional support needs

Through the family interviews, it was found that families most wanted the understanding and respect of their family, friends and people around them. They hoped to optimize the social culture of organ donation and gain social respect.

*D1: I hope to be understood and respected by society.*

*D3: I most need respect and understanding from family and friends. I’m afraid of others’ comments.*

*D4: I don’t regret organ donation, but people’s words are fearful. I am not understood by others. The community’s help after donation does not meet expectations.*

*D9: I suffered a psychological burden due to traditional thinking and the pressure of public opinion.*

*D11: Our greatest fear is to be misunderstood by others as selling organs.*

**Theme 3: Mental emotion**

(1) Positive emotions

In the family interviews, seven families (D1, D2, D3, D6, D9, D10, and D12) believed that after donating organs, their loved ones lived in another way, and their lives were extended. At the same time, they felt gratified because they contributed to society and saved others’ lives.

*D1: Donation is a good deed for others and saves lives.*

*D2: The Red Cross is the first that comes to mind. I thought there was still something alive in others; there was trust and thought in my heart. We don’t regret donating. We are happy to contribute to society. We feel comfortable giving help to people in need.*

*D8: We saw on TV many terminally ill patients dying while waiting for organ transplants and were deeply moved to learn about organ donation! We thought that there might be many families suffering from the same misfortune as ours. It would be a great thing if we could donate our daughter’s organs to save more people and to make other families happy. At the same time, it could keep our daughter alive with us forever.*

*D9: I don’t regret donating organs, it’s good to contribute to society now. It’s the continuation of my daughter’s life by another way to exist in this world.*

*D12: There was an organ donor in our village who knew something about organ donation, so we entrusted his family to contact the staff of the Red Cross Organ Donation Office, thinking that when my brother’s life can no longer continue, we hope to donate his body’s useful organs to people in need.*

(2) Negative emotions

Through the interviews, it was found that the family members also had a series of negative emotions due to traditional beliefs or lack of understanding from the people around them, such as guilt, regret, sadness, anger, and avoidance.

*D1: There are always people who say that we love money. Those who are educated will understand donation, and those who do not will talk nonsense that donation is to make money. We are so angry.*

*D5: After all, it is a very sad thing, I do not want to remember it.*

*D7: I have a little regret. Because the traditional idea is “be buried in peace.” I feel guilty.*

## Discussion

### Analysis of emotional support of organ donor families

The findings of this article show that the majority of families received emotional support and psychological comfort. In terms of public respect, most families were not sure whether the act of donation was respected by the public. The sources of emotional support given to families were mainly immediate family members, the local government and official organizations, such as the Red Cross. At the same time, the main reasons why families regretted donating (“not being understood by others,” “shame to the donor,” and “fear of people’s words”) showed that families were afraid of criticism from friends, relatives, and colleagues after donation. Gossip and public pressure can be physically and mentally exhausting. Coping style, as a mediating mechanism of mental health and stress response, play an important role in the physical and mental health of individuals (Luo A. et al., 2021). In this study, the family members with positive coping style received more emotional support than those with negative coping style.

Emotional support is a way for individuals to reduce the occurrence of mental and behavioral health problems by meeting their emotional needs and spiritual comfort through emotional compassion and empathy given by peers, family, and society (Wang et al., 2017). Stouder et al. (2009) found that family support helped the most in healing their grief, followed by support from friends, religion, and cultural beliefs. Poppe et al. (2019) showed that 88.5% of organ donor families could rely on emotional support in the first phase in the intensive care units (ICU)/emergency departments (ED), and the physician was perceived as the most active caregiver in terms of emotional support during the entire procedure. Soria-Oliver et al. (2020) found that if hospital staff members could anticipate bereaved relatives’ emotional reactions and provide better support during the grieving process, it could increase family members’ wellbeing and facilitate a better-informed organ donation decision. Luo D. et al. (2021) summarized the psychological experiences of organ donor families during the organ donation process and suggested giving psychological support to the families of organ donors. Yang et al. (2016) explained the psychological harm suffered by the family members of organ donors, as well as the steps and objectives of psychological assistance for them.

### Analysis of the emotional support needs of organ donor families

The majority of families expressed the need for spiritual comfort and emotional support. Family members mostly need the emotional support of their relatives. The support and help provided by the family in physical, emotional, and social aspects are essential (Sque et al., 2018). From the interviews, it could be seen that many family members had different degrees of psychological stress due to traditional beliefs and the pressure of public opinion. Therefore, they preferred to be understood by the people around them and the public. The research team (Huang et al., 2019) found that the concept of a “complete corpse” and public opinion were important factors influencing the decisions of donor families. Therefore, strengthening organ donation publicity and improving the transparency of organ donation are conducive to creating a favorable organ donation atmosphere (Kim et al., 2019).

Through the interviews, it was found that organ donor families wished that the state would establish professional psychological support institutions for them. However, at this stage in China, mental health professionals are in short supply. It is difficult to carry out face-to-face mental health interventions for donor family members (Pan et al., 2021). Some nurses or coordinators provide bereavement care to the relatives of organ donors during their stay in the hospital (Wang et al., 2016; Yang et al., 2017; Xie, 2020). In recent years, social workers and volunteers have gradually become important in the psychological assistance of organ donation families in big cities of China (Pan, 2019). However, it still lacks specialization and cannot be popularized for the relatives of donors to provide psychological support education.

In April, the World Health Organization (2019) released the world’s first guide to digital health interventions. It presented recommendations on 10 ways for countries to use digital health technologies to improve human health and basic services (2019). The effectiveness of digital mental health interventions (DMHIs) has also been confirmed by numerous studies (Valentine et al., 2020; Rauschenberg et al., 2021). Therefore, as smartphones become an important channel for the public to access health information and medical services, the establishment of a DMHI platform for donor families is a more implementable path.

### Theoretical implications

Family members play a prominent role in the eventual organ donation decision. At present, there are many studies on family consent and the influencing factors in the decision-making process (Lopez et al., 2018; Pan et al., 2021; Rafii and Rahimi, 2022). And there are some literature about organ donor families’ psychological experiences during the making decision (Luo A. et al., 2021; Bjelland and Jones, 2022). Previous studies focused on emotional support for relatives during the donation procedure (Mills and Koulouglioti, 2016; Poppe et al., 2019; Ahmadian et al., 2020; Ma et al., 2021). However, the experimental and theoretical research on psychological support for donor families after the donation is still few, and the study is no-depth. Kim et al. (2014) found that nearly a quarter (24.1%) of those interviewed stated they had not yet overcome their suffering, so continuous and systematic support is needed to promote the relatives’ psychological stability. This study, through questionnaire survey and qualitative interview, identified the emotional support needs of the family members of organ donors, which are beneficial to prevent and alleviate the psychological harm of organ donors’ families after donation, and provide reference to potential improvements in public health, educational, and health system levels.

### Limitations

The family members of organ donors are a special group, and it is difficult to conduct a cross-sectional study with them. Therefore, the sample size of this study was limited. In addition, we developed a self-administered Emotional Support Questionnaire for Families of Organ Donors, which was revised after expert discussion and a pre-survey. The questionnaire entries are relatively simple and may not cover all information about the emotional support obtained and needed by donor families. Thus, we conducted a mixed-methods research with combination of qualitative and quantitative methods to compensate for this deficiency to some extent. In this article, we mainly investigate the emotional support, influencing factors, and needs of the family members of organ donors, but no intervention. Thus, in future research, we will focus on psychological intervention or psychological education for the families of organ donors.

## Conclusion

Most organ donor families received emotional support from family, Red Cross, and friends, but only a minority of donor families reported receiving respect from the public after donation. Lack of understanding by others and shame for the donor were the main reasons why donor families regretted their decisions to donate. Studies have shown that marital status, occupation, health status, and coping style can affect the emotional support of donor families. The survey found that donor families showed a strong need for emotional support and hoped that the state would establish professional psychological aid institutions for donor families.

## Data availability statement

The original contributions presented in this study are included in the article/supplementary material, further inquiries can be directed to the corresponding author.

## Ethics statement

The studies involving human participants were reviewed and approved by the Institutional Review Board of The Third Xiangya Hospital, Central South University (No. 2016-S257). The patients/participants provided their written informed consent to participate in this study.

## Author contributions

PH participated in research design and data analysis. SK participated in data analysis and manuscript writing. HH and HX collected data and did the literature search. WX participated in the research design, data analysis, and manuscript writing. All authors approved the final submission.

## References

[B1] Ahmadian S. Khaghanizadeh M. Khaleghi E. Zarghami M. H. Ebadi A. (2020). Stressors experienced by the family members of brain-dead people during the process of organ donation: A qualitative study. *Death Stud.* 44 759–770. 10.1080/07481187.2019.1609137 31058581

[B2] Bjelland S. Jones K. (2022). A systematic review on improving the family experience after consent for deceased organ donation. *Prog. Transpl.* 32 152–166. 10.1177/15269248221087429 35491690

[B3] Chen C. (2021). *Tributes are also needed for the families of organ donors.* Quzhou: Quzhou Daily.

[B4] China Organ Donation Administrative Center (2022). *Policies and regulations [Online].* Available online at: http://www.codac.org.cn (accessed March 01, 2022).

[B5] Dai X. Y. (2010). *Handbook of common psychological assessment scales.* Beijing: People’s Army Medical Publishing House.

[B6] Hong Q. N. Pluye P. Fabregues S. Bartlett G. Boardman F. Cargo M. (2019). Improving the content validity of the mixed methods appraisal tool: A modified e-Delphi study. *J. Clin. Epidemiol.* 111 49–59. 10.1016/j.jclinepi.2019.03.008 30905698

[B7] Huang P. Luo A. Xie W. Xu Z. Li C. (2019). Factors influencing families’ decision-making for organ donation in Hunan Province, China. *Transpl. Proc.* 51 619–624. 10.1016/j.transproceed.2019.01.052 30979443

[B8] Jiang W. S. Sun Y. Yan J. Jiang F. Wang H. Ma Q. (2021). Overview of global organ donation and transplantation in 2020. *Organ Transpl.* 12 376–383. 10.3969/j.issn.1674-7445.2021.04.002

[B9] Kim H. S. Yoo Y. S. Cho O. H. (2014). Satisfaction with the organ donation process of brain dead donors’ families in Korea. *Transpl. Proc.* 46 3253–3256. 10.1016/j.transproceed.2014.09.094 25498033

[B10] Kim S. Sin S. M. Lee H. Y. Park U. J. Kim H. T. Roh Y.-N. (2019). Survey for the opinion of medical students and medical staff on a financial incentive system for deceased organ donation in an Asian country. *Transpl. Proc.* 51 2508–2513. 10.1016/j.transproceed.2019.04.077 31473008

[B11] Knhis N. d. S. Martins S. R. Pestana Magalhaes A. L. Ramos S. F. Sell C. T. Koerich C. (2021). Family interview for organ and tissue donation: Good practice assumptions. *Rev. Bras. Enferm.* 74:e20190206. 10.1590/0034-7167-2019-0206 34161535

[B12] Li M. T. Hillyer G. C. Husain S. A. Mohan S. (2019). Cultural barriers to organ donation among Chinese and Korean individuals in the United States: A systematic review. *Transpl. Int.* 32 1001–1018. 10.1111/tri.13439 30968472 PMC6867085

[B13] Lopez J. S. Soria-Oliver M. Aramayona B. Garcia-Sanchez R. Martinez J. M. Martin M. J. (2018). An integrated psychosocial model of relatives’ decision about deceased organ donation (IMROD): Joining pieces of the puzzle. *Front. Psychol.* 9:408. 10.3389/fpsyg.2018.00408 29692744 PMC5902731

[B14] Luo A. He H. Xu Z. Deng X. Xie W. (2021). Social support of organ donor families in China: A quantitative and qualitative study. *Front. Public Health* 9:746126. 10.3389/fpubh.2021.746126 34869161 PMC8637885

[B15] Luo D. Chen J. Liao L. Xie Q. Wu J. (2021). Qualitative research on decision-making experience of family members of Chinese organ donation after citizen’sdeath. *Chin. Evid. Based Nurs.* 7 1209–1213. 10.12102/j.issn.2095-8668.2021.09.014

[B16] Ma J. Zeng L. Li T. Tian X. Wang L. (2021). Experiences of families following organ donation consent: A qualitative systematic review. *Transpl. Proc.* 53 501–512. 10.1016/j.transproceed.2020.09.016 33483168

[B17] Mills L. Koulouglioti C. (2016). How can nurses support relatives of a dying patient with the organ donation option? *Nurs. Crit. Care* 21 214–224. 10.1111/nicc.12183 25943336

[B18] Nie J.-B. Jones D. G. (2019). Confucianism and organ donation: Moral duties from xiao (filial piety) to ren (humaneness). *Med. Health Care Philos.* 22 583–591. 10.1007/s11019-019-09893-8 30903406

[B19] Pan X. T. Ma J. Liu W. Bai Z. C. Dai Z. F. Huang J. T. (2021). Investigation and strategic analysis of family barriers to organ donation in China. *Transpl. Proc.* 53 513–519. 10.1016/j.transproceed.2020.09.017 33293039

[B20] Pan Y. (2019). Models of family assistance after organ donation and social workers intervention with the perspective of the crisis intervention theory. *Chin. Med. Ethics* 32 1294–1297.

[B21] Peng B. Xu J. Zen M. Pan Q. Shi H. Jiang J. (2021). From medicine to society: A cultural turn of significance for organ donation brought by a major epidemic. *Chin. Med. Ethics* 34 675–679.

[B22] Poppe C. Akum S. Crombez G. Rogiers X. Hoste E. (2019). Evaluation of the quality of the communication and emotional support during the donation procedure: The use of the donor family questionnaire (DFQ). *J. Crit. Care* 53 198–206. 10.1016/j.jcrc.2019.06.011 31271955

[B23] Rafii F. Rahimi S. (2022). Organ donation decision in families with brain-dead patients an evolutionary concept analysis. *Prof. Case Manag.* 27 67–84. 10.1097/ncm.0000000000000529 35099421

[B24] Rauschenberg C. Schick A. Hirjak D. Seidler A. Paetzold I. Apfelbacher C. (2021). Evidence synthesis of digital interventions to mitigate the negative impact of the COVID-19 pandemic on public mental health: Rapid meta-review. *J. Med. Internet Res.* 23:e23365. 10.2196/23365 33606657 PMC7951054

[B25] Schoonenboom J. Johnson R. B. (2017). How to construct a mixed methods research design. *Kolner Z. Soziol. Sozialpsychol.* 69 107–131. 10.1007/s11577-017-0454-1 28989188 PMC5602001

[B26] Shi B. Y. Liu Z. J. Yu T. (2020). Development of the organ donation and transplantation system in China. *Chin. Med. J.* 133 760–765. 10.1097/cm9.0000000000000779 32195670 PMC7147652

[B27] Soria-Oliver M. Aramayona B. Lopez J. S. Martin M. J. Martinez J. M. Saenz R. (2020). Grief reactions of potential organ donors’ bereaved relatives: An observational study. *Am. J. Crit. Care* 29 358–368. 10.4037/ajcc2020960 32869074

[B28] Sque M. Walker W. Long-Sutehall T. Morgan M. Randhawa G. Rodney A. (2018). Bereaved donor families’ experiences of organ and tissue donation, and perceived influences on their decision making. *J. Crit. Care* 45 82–89. 10.1016/j.jcrc.2018.01.002 29413728

[B29] Stouder D. B. Schmid A. Ross S. S. Ross L. G. Stocks L. (2009). Family, friends, and faith: How organ donor families heal. *Prog. Transpl.* 19 358–361. 10.1177/152692480901900412 20050460

[B30] Tirgari B. Samareh H. Forouzi M. A. (2020). Relationship between bereavement reaction with posttraumatic stress disorder and depression in organ donor families in Iran. *J. Neurosci. Nurs.* 52 21–26. 10.1097/jnn.0000000000000486 31842030

[B31] Valentine L. McEnery C. Bell I. O’Sullivan S. Pryor I. Gleeson J. (2020). Blended digital and face-to-face care for first-episode psychosis treatment in young people: Qualitative study. *JMIR Ment. Health* 7:e18990. 10.2196/18990 32720904 PMC7420518

[B32] Wang L. Lv Y. Tian M. Jia L. Zhang X. Shi J. (2016). Analysis on the application of grief counseling in alleviating the grief of organ donor families. *Chin. Med. Ethics* 29 630–632. 10.12026/j.issn.1001-8565.2016.04.26

[B33] Wang Y. F. Reitter D. Yen J. (2017). “How emotional support and informational support relate to linguistic alignment,” in *Proceedings of the 10th International conference on social, cultural, and behavioral modeling and prediction and behavior representation in modeling and simulation (SBP-BRiMS))*, Washington, DC, 25–34.

[B34] Wind T. Jansen N. Floden A. Haase-Kromwijk B. Shaw D. Gardiner D. (2022). An inventory of deceased donor family care and contact between donor families and recipients in 15 European Countries. *Transpl. Int.* 35:10188. 10.3389/ti.2021.10188 35185370 PMC8842228

[B35] World Health Organization (2019). *WHO guideline: Recommendations on digital interventions for health system strengthening.* Geneva: World Health Organization. 31162915

[B36] Xie Q. (2020). The relieving effect of grief counseling combined with standardized communication on the grief of organ donor families. *Home Med.* 6 377–378.

[B37] Xie W. Li C. (2016). Analysis of factors influencing decision making of families of minor and adult organ donors based on semi-structured interview method. *Chin. J. Transpl.* 10 126–129.

[B38] Xie Y. (1998). A preliminary study on the reliability and validity of the simplified coping style scale. *Chin. J. Clin. Psychol.* 6 53–54.

[B39] Yang C. Yin L. Feng L. Bai L. Wang L. (2017). Grief nursing care of organ donor family members. *Prac. J. Organ. Transpl.* 5 140–141. 10.3969/j.issn.2095-5332.2017.02.012

[B40] Yang S. Huang L. Tan J. (2016). Psychological assistance after organ donation. *Organ Transpl.* 7 89–93.

[B41] Yang X.-W. Xiong T.-W. Hua X.-F. Xu Q. Tang Y.-E. Chen W.-J. (2018). Anxiety and depression among families of deceased donors in China. *Chin. Med. J.* 131 99–102. 10.4103/0366-6999.221278 29271388 PMC5754966

[B42] Zhang X. Feng L. (2019). Using psychological crisis intervention to alleviate the cultural sensitivity of organ donation in China. *Organ Transpl.* 10 84–87. 10.3969/j.issn.1674-7445.2019.01.013

